# Interaction between NSMCE4A and GPS1 links the SMC5/6 complex to the COP9 signalosome

**DOI:** 10.1186/s12860-020-00278-x

**Published:** 2020-05-08

**Authors:** András Horváth, Gergely Rona, Michele Pagano, Philip W. Jordan

**Affiliations:** 1 Biochemistry and Molecular Biology Department, Johns Hopkins University Bloomberg School of Public Health, Baltimore, MD 21205 USA; 2 Department of Biochemistry and Molecular Pharmacology, New York University School of Medicine, New York, NY 10016 USA; 3 Perlmutter Cancer Center, New York University School of Medicine, New York, NY 10016 USA; 4 Howard Hughes Medical Institute, New York University School of Medicine, New York, NY 10016 USA

**Keywords:** NSMCE4A, SMC5/6, GPS1, COP9 signalosome, DNA repair, Deneddylation

## Abstract

**Background:**

The SMC5/6 complex, cohesin and condensin are the three mammalian members of the structural maintenance of chromosomes (SMC) family, large ring-like protein complexes that are essential for genome maintenance. The SMC5/6 complex is the least characterized complex in mammals; however, it is known to be involved in homologous recombination repair (HRR) and chromosome segregation.

**Results:**

In this study, a yeast two-hybrid screen was used to help elucidate novel interactions of the kleisin subunit of the SMC5/6 complex, NSMCE4A. This approach discovered an interaction between NSMCE4A and GPS1, a COP9 signalosome (CSN) component, and this interaction was further confirmed by co-immunoprecipitation. Additionally, GPS1 and components of SMC5/6 complex colocalize during interphase and mitosis. CSN is a cullin deNEDDylase and is an important factor for HRR. Depletion of GPS1, which has been shown to negatively impact DNA end resection during HRR, caused an increase in SMC5/6 levels at sites of laser-induced DNA damage. Furthermore, inhibition of the dennedylation function of CSN increased SMC5/6 levels at sites of laser-induced DNA damage.

**Conclusion:**

Taken together, these data demonstrate for the first time that the SMC5/6 and CSN complexes interact and provides evidence that the CSN complex influences SMC5/6 functions during cell cycle progression and response to DNA damage.

## Background

Eukaryotes express three classes of structural maintenance of chromosome (SMC) complexes; cohesin, condensin and SMC5/6. Each SMC complex is comprised of two large SMC ATPases, a conserved kleisin and other accessory proteins [1, 2]. The SMC proteins have two extensive coiled-coil domains interrupted by a hinge domain that folds each SMC back on itself. The two globular C and N terminal ends are juxtaposed to form an ATP-binding and ATP-hydrolysis site. Interaction between two SMC hinge domains forms a V-shaped heterodimer, which is closed to form a ring-like structure by the kleisin subunit.

The canonical function for cohesin is to hold sister chromatids together from S-phase until the onset of anaphase [3]. Condensin complexes are best known for their role in chromatin condensation prior to chromosome segregation [4]. Although much less research has been published about the SMC5/6 complex it is known to be involved in DNA damage repair and chromosome segregation during mitosis and meiosis [1, 5–11].

NSMCE4A (non-SMC element 4A) is the kleisin subunit of the SMC5/6 complex that bridges the ATPase head/tail domains of SMC5 and SMC6 [12]. Previous studies have shown that NSMCE4A directly interacts with two other components of the SMC5/6 complex, an E3 ubiquitin ligase, NSMCE1, and a MAGE (melanoma-associated antigen gene) domain containing protein, NSMCE3 [12–14].

In this study, a yeast two-hybrid screen was performed to identify whether NSMCE4A directly interacts with additional proteins, beyond the SMC5/6 complex components. It was determined that NSMCE4A interacts with GPS1 (G protein pathway suppressor 1; also known as CSN1 and COPS1). GPS1 is a component of an eight-subunit complex called the COP9 (constitutive photomorphogenesis 9) signalosome (CSN) complex [15]. From further interaction and localization analyses, we obtained evidence that the interaction between NSMCE4A and GPS1 involves the entire CSN and SMC5/6 complexes.

CSN contains two MPN (MPR1/PAD1 amino-terminal) domain-containing proteins (CSN5 and CSN6) and six different PCI (proteasome lid-CSN- initiation factor 3) proteins (GPS1, CSN2–CSN4, CSN7 and CSN8) [15, 16]. The primary function of CSN is to inactivate Cullin–RING E3 ubiquitin ligases (CRLs) by enzymatically removing their ubiquitin-like activator, NEDD8 [17]. CSN5 harbors the enzymatic activity of cullin deNEDDylation. CSN is regulated by autoinhibition, and is only active when bound to neddylated CRLs [15, 16]. CRLs and CSN are involved in many cellular processes including cell cycle regulation, DNA replication, development, transcriptional regulation, and protein quality control [18]. One of the main functions of CRLs is to regulate the DNA damage response (DDR), and CSN regulates CRLs activity at sites of DNA damage [19–26]. Additionally, the abrogation of CSN function has been shown to affect chromosome segregation during mitosis and meiosis [27–31].

As the SMC5/6 and CSN complexes have functional overlap with regards to DNA damage response and chromosome segregation, we assessed whether alteration of cullin NEDDylation status influences SMC5/6 localization during cell proliferation and following DNA damage.

## Results

### Yeast two-hybrid screening for interacting partners of NSMCE4A

Mouse prey cDNA library was tested with NSMCE4A bait in yeast two-hybrid screening. From the positive interacting clones, 40 clones were subsequently isolated and sequenced. To minimize false-positive interactions, the yeast two-hybrid selection conditions were repeated. This reduced the number of positive clones to 28, that represented 16 genes. (Supplemental Table S1). Some genes were represented by multiple cDNA clones differing in fragment size. For example, GPS1 was represented by three cDNA sequences of differing size. The focus of this study was the characterization of the interaction between NSMCE4A and GPS1.

### NSMCE4A interacts with GPS1 via yeast two-hybrid and co-immunoprecipitation

**Fig. 1:**
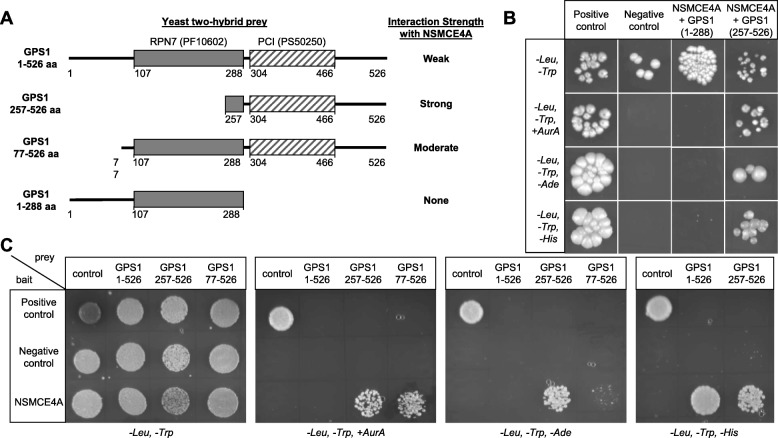
Characterization of the interaction between NSMCE4A and GPS1 by yeast-two-hybrid experiments. (**a**) Schematic of each GPS1 cDNA prey construct used in the yeast-two hybrid experiments assessing their binding to NSMCE4A. Full length GPS1 is 526 amino acids (aa) long. The schematics include two conserved domains, the RPN7 homology box (PF10602) and the proteasome component (PCI) domain (PS50250). Strength of interaction between each GPS1 prey and NSMCE4A bait is summarized on the right of each prey diagram. (**b**) Yeast two hybrids grown on a series of selection media to assess interaction between full length NSMCE4A bait and an empty prey vector (negative control), truncated GPS1 prey that cover the N-terminal region (GPS1, 1–288 aa), and C-terminal region (GPS1, 257–526 aa). NSMCE4A bait and GPS1 prey constructs were tested in parallel with a positive bait and prey control (see materials and methods). The media *-Leu-Trp* does not contain any selection for the interaction. The interaction was tested via the expression from the cassettes encoding Aureobasidin A resistance (*AurA*), Adenine (*−Ade*) or Histidine (*−His*) synthetases. (**c**) Yeast two hybrid interaction results between full length NSMCE4A bait and specified GPS1 constructs (GPS1, 1–526 aa; GPS1, 257–526 aa; GPS1, 77–526 aa). NSMCE4A bait and GPS1 prey constructs were tested in parallel with a positive and negative bait and prey controls (see materials and methods)

GPS1 has two defined conserved motifs (Fig. 1a), the 26S Proteasome RPN7 homology motif (107 to 288 aa), and the Proteasome, COP9, Initiation factor 3 (PCI) motif (304–466 aa). In total, four prey constructs that encoded different lengths of the GPS1 cDNA were screened for interaction with a full length NSMCE4A prey via yeast two-hybrid (Fig. 1a). Interactions were examined for growth on three selection conditions: aureobasidin A supplemented, adenine drop-out, and histidine drop-out media (Fig. 1b and c). Aureobasidin A is the most stringent condition, while selection for growth on histidine is the least stringent due to possible residual *HIS3* gene expression [32]. When co-expressed with the NSMCE4A bait plasmid, the prey plasmid encoding for the full-length sequence of GPS1 (GPS1, 1–526 amino acids, aa) only grew on the least stringent histidine dropout selection condition (Fig. 1c). In contrast, a strong interaction between NSMCE4A and GPS1 was observed when only the C-terminal half, containing the PCI motif, was present (GPS1, 257–526 aa, Fig. 1b and c). Interestingly, compared to the full length GPS1, the interaction between GPS1 and NSMCE4A was stronger when the first 77 amino acids were removed (i.e. GPS1, 77–526 aa had a higher binding affinity to NSMCE4A compared to GPS1, 1–526 aa), suggesting that the N-terminus has a negative impact on the interaction. Finally, no interaction was detected between NSMCE4A and GPS1 when the C-terminal half, containing the PCI motif, was completely absent (GPS1, 1–288 aa; Fig. 1b).

**Fig. 2:**
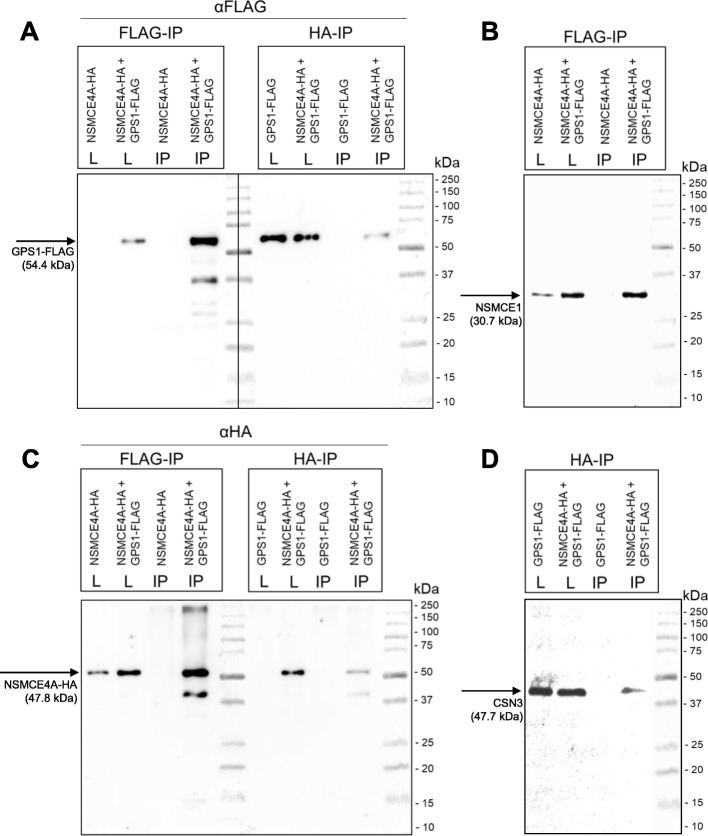
Characterization of the interaction between NSMCE4A and GPS1 by co-immunoprecipitation. HA tagged NSMCE4A and FLAG tagged GPS1 were expressed or co-expressed in HEK cells and precipitated with anti-HA or anti-FLAG antibodies. (**a**) NSMCE4A, GPS1 were copurified in FLAG-specific immunoprecipitation. (**b**) NSMCE1 was also detected in the immunoprecipitation for FLAG tagged GPS1. (**c**) NSMCE4A, GPS1 were copurified in HA-specific immunoprecipitations (**d**) CSN1 was also detected in the immunoprecipitation for HA tagged NSMCE4A. FLAG-IP: FLAG-specific immunoprecipitation, HA-IP: HA-specific immunoprecipitation, L: lysate (10.5%), IP: immunoprecipitated fraction

The interaction between NSMCE4A and GPS1 was tested by co-immunoprecipitation. FLAG-tagged mouse GPS1 and HA-tagged mouse NSMCE4A were co-expressed in HEK cells and immunoprecipitation with FLAG or with HA antibodies resulted in detecting interaction between GPS1 and NSMCE4A with no background affinity (Fig. 2). Furthermore, these interactions appear to occur within the context of the CSN and SMC5/6 complexes, as NSMCE1 was detected in the elution of the FLAG immunoprecipitation, and CSN3 was detected in the elution of the HA immunoprecipitation.

In conclusion, two independent methods confirmed the physical interaction between GPS1 and NSMCE4A, and this interaction likely occurs within the context of the entire SMC5/6 and CSN complexes.

### CSN and SMC5/6 complexes have similar localization patterns

**Fig. 3:**
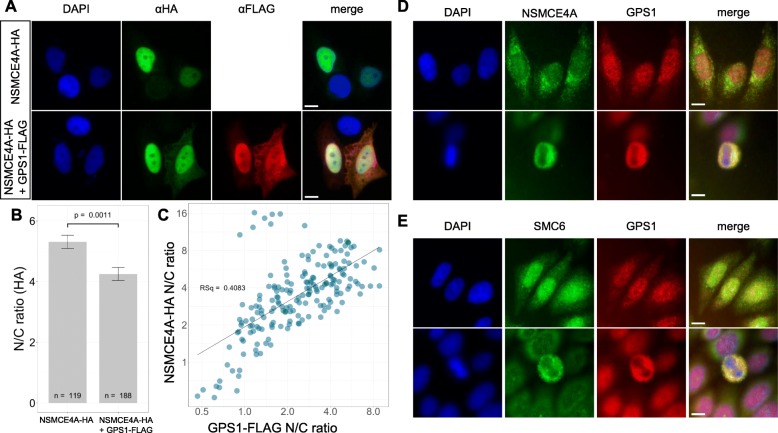
Colocalization between GPS1 and SMC5/6 components. (A-C) Co-localization of HA-tagged NSMCE4A and FLAG-tagged GPS1 in HeLa cells. (**a**) NSMCE4A localizes predominantly in the nucleus. NSMCE4A and GPS1 colocalize in the nucleus and in some cases in the cytoplasm as well, indicating, that NSMCE4A tends to localize in the cytoplasm in the presence of GPS1. (**b**) Nucleocytoplasmic distribution of NSMCE4A in the absence and presence of FLAG-tagged GPS1. NSMCE4A tends to be more nuclear if GPS1 is not overexpressed. Error bars indicate the standard error of mean (SEM). The *p*-value of t-test is indicated. The p-value of non-parametric Mann-Whitney test is 5.41 × 10^− 6^. (**c**) Relationship between the nucleocytoplasmic ratio of GPS1 and that of NSMCE4A. The plot indicates that the lower nucleocytoplasmic ratio of GPS1 corresponds to lower nucleocytoplasmic ratio of NSMCE4A. Pearson correlation coefficient: 0.639, Spearman correlation coefficient: 0.655. Colocalization of (**d**) NSMCE4A and (**e**) SMC6 with GPS1 during interphase and metaphase using antibodies raised against the endogenously expressed proteins. Size bars = 10 μm. See Supplemental Figure S1 for complementary data

The localization and co-localization of the HA tagged mouse NSMCE4A and the FLAG-tagged mouse GPS1 was assessed in HeLa cells. NSMCE4A-HA localized predominantly in the nucleus (Fig. 3a). When NSMCE4A and GPS1 were coexpressed they co-localized primarily in the nucleus. However, in cases where GPS1 staining was detected in the cytoplasm, an increased signal of NSMCE4A was observed in that compartment as well (Fig. 3a). The nucleus to cytoplasm ratio of NSMCE4A signal significantly decreased in the co-transfected cells, indicating that the presence of the recombinant GPS1 protein alters the nucleocytoplasmic distribution of NSMCE4A (Fig. 3b). Furthermore, Fig. 3c shows the linear relationship between the signal of GPS1 and NSMCE4A nucleus to cytoplasm ratios. These observations indicate a strong interaction between NSMCE4A and GPS1 in intracellular conditions. The co-localization of GPS1 and NSMCE4A and the increased retention of NSMCE4A within the cytoplasm in the presence of GPS1 suggests that the physical interaction of NSMCE4A with GPS1 hinders its nuclear import.

Colocalization between endogenously expressed GPS1 and NSMCE4A in HeLa cells was assessed using antibodies raised against each protein (Fig. 3d). GPS1 and NSMCE4A were observed within the nucleus and cytoplasm of interphase cells. At metaphase stage, GPS1 and NSMCE4A colocalized together surrounding condensed chromosomes. We also showed that NSMCE4A colocalizes with the enzymatic component of CSN, CSN5 (Supplemental Figure S1A and B). To determine whether these colocalization patterns were observed for another component of the SMC5/6 complex, antibodies raised against SMC6 were used in conjunction with the GPS1 antibody. As was the case for NSMCE4A, SMC6 colocalized with GPS1 within the nucleus and cytoplasm at interphase and around condensed chromosomes at metaphase (Fig. 3e). We also compared the localization of NSMCE1 and all eight CSN components (CSN1–8), which demonstrated the same localization patterns described for NSMCE4A, SMC6, and GPS1 (Supplemental Figure S1C-J). Depletion of GPS1 via siRNA or inhibition of CSN5 did not result in a change in localization pattern of NSMCE4A, suggesting that CSN presence or function is not required for SMC5/6 localization pattern during cell proliferation (Supplemental Figure S1K-N).

### GPS1 depletion increases SMC6 signal at sites of laser-induced DNA damage

**Fig. 4:**
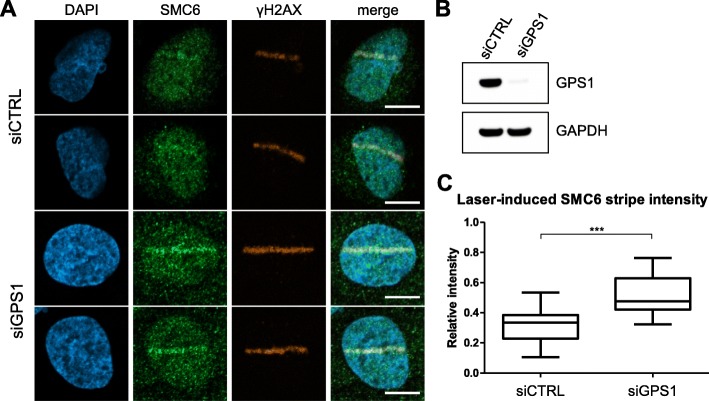
GPS1 depletion resulted in increased levels of SMC6 localization to laser induced DNA damage. (**a**) Representative images of cells exposed to laser-induced DNA damage following treatment with control siRNA or GPS1 siRNA. (**b**) Western blot assessment of GPS1 protein depletion following siRNA treatment. (**c**) Box and whiskers plot showing the quantification of SMC6 signal intensity at the site of laser-induced DNA damage compared to average signal intensity within the undamaged regions of the nucleus following treatment with control siRNA or GPS1 siRNA. *P*-value of 0.0006 was determined from a two-tailed Mann Whitney t-test. Whiskers represent the minimum and maximum measurements. Ends of the box are the upper and lower quartiles, and the median is marked by a vertical line inside the box. Size bars = 10 μm

The COP9 signalosome complex (CSN) and SMC5/6 complex components localize to sites of DNA damage including those induced by laser [11, 19]. It is also known that GPS1 depletion via siRNA results in the depletion of other CSN subunits [19]. Localization of SMC6 to laser-induced DNA damage following GPS1 siRNA-mediated depletion was assessed (Fig. 4 a and b). SMC6 signal intensity at sites of laser-induced DNA damage was increased significantly when GPS1 was depleted (Fig. 4c, Supplemental Figure S2A and B).

### Inhibition of CSN increases SMC6 signal at sites of laser-induced DNA damage

**Fig. 5:**
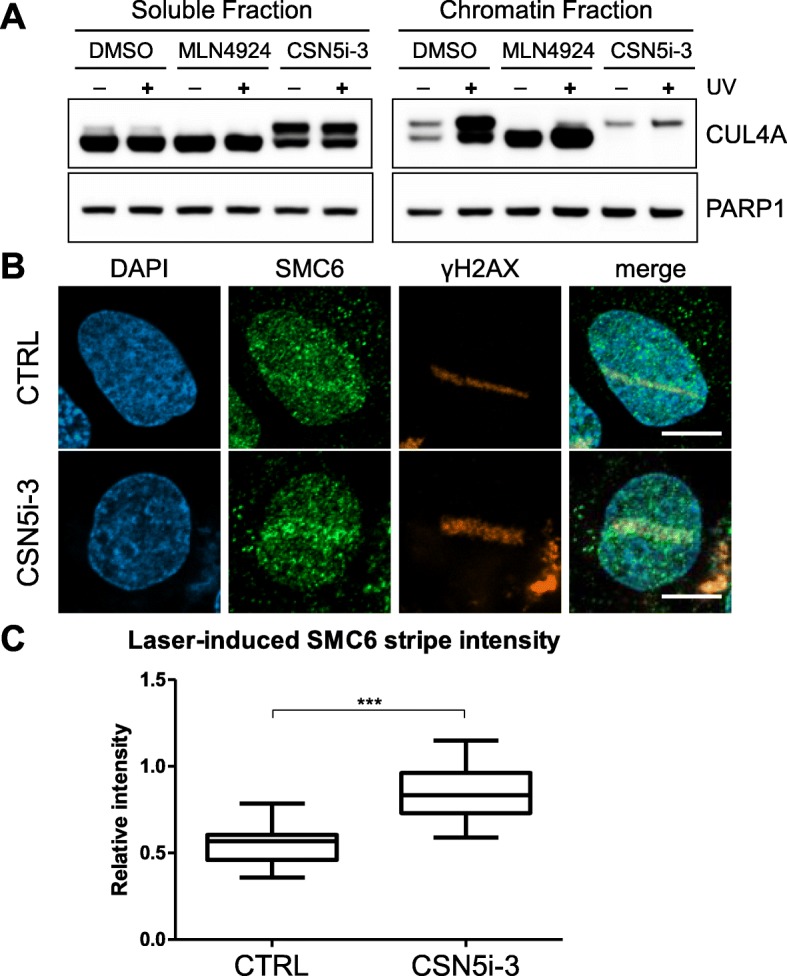
Inhibition of deneddylation function of the CSN complex resulted in increased levels of SMC6 localization to laser induced DNA damage. (**a**) Western blot assessing proteins within the soluble and chromatin bound fractions extracted from cells treated with vehicle control (DMSO), MLN4924 (neddylation inhibitor) and CSN5i-3 (deneddylation inhibitor). A portion of cells from each condition were exposed to UV radiation (75 J/m^2^) for 30 min to enhance chromatin recruitment of the CUL4 complex. (**b**) Representative images of cells exposed to laser-induced DNA damage following treatment with vehicle control or CSN5i-3. (**c**) Box and whiskers plot showing the quantification of SMC6 signal intensity at the site of laser-induced DNA damage compared to average signal intensity within the undamaged regions of the nucleus following treatment with vehicle control or CSN5i-3. P-value of < 0.0001 was determined from a two-tailed Mann Whitney t-test. Whiskers represent the minimum and maximum measurements. Ends of the box are the upper and lower quartiles, and the median is marked by a vertical line inside the box. Size bars = 10 μm

The small molecule inhibitor, CSN5i-3, was used to test whether the inhibition of cullin deneddylation by the CSN was influencing SMC6 localization to sites of laser-induced DNA damage. The inhibition of CSN using CSN5i-3 was confirmed by assessing the neddylation status of CUL4A, a primary target of CSN-mediated deneddylation, in the presence and absence of DNA damage (Fig. 5a). The upper band of CUL4A corresponds to the neddylated form, which is enriched upon treatment with CSN5i-3 and is decreased when applying the NEDD8 E1 inhibitor, MLN4924. This could be observed both in the soluble fraction and the chromatin enriched fraction of CUL4A following UV treatment (Fig. 5a). SMC6 signal intensity at sites of laser-induced DNA damage was increased significantly when the CSN activity was inhibited by with CSN5i-3 (Fig. 5b and c, Supplemental Figure S2C and D).

## Discussion

### Links between the SMC5/6 and CSN complexes

This study marks the first focused study that physically and functionally links the SMC5/6 complex and CSN complex together in the cell. There are some previously reported data from high-throughput analyses that link the CSN and SMC5/6 complexes. A study using CRISPR to systematically perturb 222,784 gene pairs in two cancer cells lines determined a genetic interaction between *SMC5* and a CSN component, *COPS4*, which resulted in a growth abnormality [33]. Using similar high-throughput genetic interaction approaches in *Schizosaccharomyces pombe* and *Saccharomyces cerevisiae* it has been comprehensively shown that increased growth defects result when a mutation in one of the components of the SMC5/6 complex is combined with a mutation in one of the components of the CSN complex [34–37]. Components of the CSN and SMC5/6 complexes also share several physical interaction partners discovered from high-throughput interaction analyses, which include components of the other two SMC complexes (cohesin and condensin), a component of the MIS12 kinetochore complex (PMF1) and the RECQL4 DNA repair helicase [11, 33, 38–43]. Hepatitis B virus regulatory protein X (HBx) interacts with the CRL4 (DDB1-CUL4-ROC1) E3 ubiquitin ligase and targets SMC5/6 components for degradation [44, 45]. As CSN regulates CRL4 activity, it is possible that CSN maintains SMC5/6 stability, which is a good direction for future investigations.

### The role of the SMC5/6 and CSN complexes during mitosis and meiosis

Here, it was demonstrated that SMC6, NSMCE4A and GPS1 colocalize during mitosis, with each protein surrounding the condensed chromosomes. This localization pattern has also been reported for SMC5/6 components in mouse embryonic cells (mESCs) and human retinal pigmented epithelial (RPE-1) cells [7, 46]. A similar localization pattern was also shown for COP9 component, CSN5, in HeLa cells [47]. It is possible that the interaction between the SMC5/6 and CSN complexes could influence the regulation of chromosome condensation. Depletion of SMC5/6 causes condensin localization defects during mitosis and meiosis [7, 8, 46]. As the CSN is the master regulator of all CRLs, its role during mitosis is broad, affecting many processes such cell cycle regulation, and DNA replication [16, 25, 48]. For instance, depletion of CSN components in HeLa cells and mouse oocytes demonstrated that chromosomes form abnormal aggregates and fail to segregate normally during mitosis and meiosis [28, 47]. It is possible that CRL regulation by CSN influences essential functions of the SMC5/6 complex during chromosome segregation.

### The role of the SMC5/6 and CSN complexes in DNA damage response

Upon DNA damage, components of the CSN complex are phosphorylated by the DNA damage response serine/threonine kinase, ATM (Ataxia Telangiectasia Mutated) [19, 20, 22]. These phosphorylation events are thought to facilitate efficient DNA repair. With regards to double-strand break (DSB) repair, CSN has been shown to be recruited to DSB sites, and the complex is important for DNA end resection during homologous recombination repair (HRR) [19]. The SMC5/6 complex is also important during HRR events. In mammalian cells it has been demonstrated that the E3 SUMO ligase component of the SMC5/6 complex, NSMCE2, SUMOylates multiple lysines of the kleisin subunit of cohesin, RAD21 (also known as SCC1) [11]. These NSMCE2-mediated SUMOylation events on RAD21 are required to ensure proficient HRR between sister chromatids by ensuring cohesin is stabilized around the DSB. Both the CSN complex and SMC5/6 complex localize to laser-induced DNA damage sites, and this study has demonstrated that CSN inhibition results in increased levels of SMC5/6 accumulation at sites of laser-induced DNA damage. When CSN is inhibited the SMC5/6 complex may not be able to efficiently SUMOylate targets such as cohesin, subsequently resulting in inefficient or perturbed HRR.

## Conclusions

The data presented in this study has demonstrated a solid physical and functional interaction between the SMC5/6 and CSN complexes. Going forward, it is important to establish how these two complexes are influencing DNA damage repair processes and how they relate to the global response to DNA damage. Additionally, the two complexes may be involved in overlapping functions with regards to chromosome compaction and segregation, which is another area for further exploration.

## Methods

### Plasmids used in the study

FLAG-tagged mouse GPS1 cDNA encoding vector was obtained from Origene (pCMV6-Entry DDK/Myc GPS1 transcript variant 1). Mouse NSMCE4A cDNA derived from the vector pCMV6-AC-GFP NSMCE4A (Origene) was cloned into the BamHI and XhoI sites of pCMV6-Entry vector (Origene) using BglII and XhoI. To create HA-tagged constructs, a modified pcDNA5/FRT/TO vector (Invitrogen) was used as a starting point, in which a HA tag encoding segment was cloned by KpnI and BamHI sites (5′- ggt acc gcc gcc acc atg tac cca tac gac gta cca gat tac gct gga tcc − 3′) resulting the vector pcDNA5/FRT/TO-HA. NSMCE4A cDNA and GPS1 cDNA was cloned into this vector using the primer pairs (BamHI: 5′- cag gga tcc atg tct ggc gac agc ag − 3′; XhoI: 5′- cgg cac tcg atc tcc atg g − 3′) and (BamHI: 5′- cag gga tcc atg cgg ggc agc − 3′; NotI: 5′- gat gag ttt ctg ctc gag cg − 3′) respectively. Sequence of all constructs made was tested by restriction digestion and sequencing.

### Cell cultures and transfection

HEK 293 T cells were used for western blot and co-immunoprecipitation experiments. HeLa cells were used for immunocytochemistry and localization tests. The cells were maintained in DMEM (Life Technologies) supplemented with 10% FBS (Thermo Fisher) and 1% penicillin/streptomycin. U-2 OS cells were used for laser induced DNA damage recruitment and cell fraction experiments. Cells were maintained in McCoy’s 5A medium supplemented with 10% FBS (Corning Life Sciences) and 1% penicillin/streptomycin/L-glutamine (Corning Life Sciences). All the cells were incubated at 37 °C in humidified incubator in the presence of 5% CO2. The cells were transfected with Lipofectamine 2000 (Invitrogen) as recommended by the manufacturer. RNAi silencing transfections were performed for 48 h using Lipofectamine RNAiMAX (Invitrogen) based on the manufacturer’s recommendations. ON-TARGETplus Human GPS1 (2873) siRNA SMARTpool (L-012272-00-0005; Dharmacon) was used for GPS1 depletion. MLN-4924 (Active Biochem) and CSN5i-3 (Novartis) was added 1 h prior to imaging at 2 μM final concentration and was kept on the cells during laser micro-irradiation.

### Co-immunoprecipitation and western blot

Co-immunoprecipitation was performed using Dynabeads co-immunoprecipitation kit (Life Technologies). Cell lysis was performed using the lysis buffer provided in the kit supplemented with 100 mM NaCl, 1 mM dTT and 1:25 EDTA-free protease inhibitor cocktail (Roche). The supplied elution buffer was neutralized by 100 mM TRIS after reconstitution of the immunoprecipitated fraction. For antibody immobilization 7.5 μg anti-HA antibody (GeneTex GTX628489) and 15 μg anti-FLAG antibody (Sigma F3165) was coupled to 1.5 mg magnet beads. For western blotting, lysates were mixed with 2x Laemmli Sample Buffer (Bio-Rad) and run on 12% denaturing PAGE. For western blot studies where indicated, cells were fractionated into soluble and chromatin bound protein fractions. Cells were washed with ice-cold PBS, lysed in CSK buffer for 10 min to get the soluble fraction (10 mM PIPES [pH = 7.0], 100 mM NaCl, 300 mM sucrose, 3 mM MgCl_2_, 1 mM EGTA, 1 mM DTT, 0.2% Triton X-100, cOmplete™ ULTRA protease inhibitor (Roche), PhosSTOP phosphatase Inhibitor (Roche). The pellet was resuspended in the following buffer to get the chromatin bound protein fraction: 50 mM TRIS.HCl [pH = 7.4], 250 mM NaCl, 1 mM EDTA, 3 mM MgCl_2_, 1 mM DTT, 0.2% Triton X-100 and 1 unit/ μl Bezonase (Sigma), cOmplete™ ULTRA protease inhibitor (Roche), PhosSTOP phosphatase Inhibitor (Roche). Proteins were blotted to PVDF membrane by Bio-Rad Trans-Blot Turbo Transfer System at pH = 8.3. Western blots were incubated with indicated primary then corresponding secondary antibodies (Supplemental Table S2). Uncropped membranes and western blot images are presented in Supplemental Figure S3 and S4.

### Immunocytochemistry

HeLa cells were fixed in 5% PFA in PBS or ice-cold methanol and immunostained with incubation with indicated primary then corresponding secondary antibodies (Supplemental Table S2). Cells were analyzed using Zeiss AxioImager A2 fluorescent microscope with AxioCam ERc 5 s (Zeiss) camera. The images were processed and quantified by Fiji [49]. Statistical analysis (two-tailed t-test, Mann-Whitney U test, Spearman correlation) was performed by R software or GraphPad Prism 5.

### Yeast two hybrid

Yeast-two-hybrid prey mouse cDNA library and bait mouse NSMCE4A cDNA constructs were created by Creative Biolabs United Kingdom. Clontech pGB plasmid in yeast strain Y190 was used as bait and tested for the absence of self-activation and toxicity. Mouse testis Matchmaker cDNA library (Clontech) in pACT2 vector was used as prey. Creative Biolabs found 121 interacting clones from which 41 clones were re-isolated and sequenced. Subsequent validations were performed on the isolated 41 clones using Matchmaker Y2H System (Clontech) following the instructions of the manufacturer. Yeast hybrids were selected on SD -Leu, −Trp agar media. Interaction was tested on either SD -Leu, −Trp, −Ade; SD -Leu, −Trp -His or SD -Leu, −Trp supplemented with 200 ng/ml Aureobasidin A (Clontech).

### Laser-induced DNA damage and imaging

Laser-induced DNA damage induction was performed as in [50]. Following RNA silencing, ~ 80.000 U-2 OS cells were plated per well of a four-well Lab-Tek II chambered coverglass 24 h before imaging. Cells were imaged in FluoroBrite™ DMEM supplemented with 10% FBS, 25 mM HEPES, and sodium pyruvate. Laser induced laser stripes were done on a Zeiss LSM 800 microscope, using a 405 nm diode laser (5 mW) with the timed bleach option (60 iterations, 80% laser power output) in the ZenBlue 2.1 software using a Plan-Apochromat 63x/1.40 Oil objective after pre-sensitizing the cells with 1 μg/ml Hoechst 33342 (Molecular probes) for 30 min. 10 min after irradiation, cells were washed with ice-cold CSK extraction buffer (10 mM PIPES [pH = 7.0], 100 mM NaCl, 300 mM Sucrose, 3 mM MgCl_2_, 1 mM EGTA, 0.2% Triton X-100, cOmplete™ ULTRA protease inhibitor (Roche), PhosSTOP phosphatase inhibitor (Roche)) for 5 min and were subsequently fixed in 4% PFA in PBS for 15 min at room temperature. Samples were then blocked in blocking buffer (PBS containing 0.05% Triton X-100, 5% FBS and 3% BSA) before incubation with indicated primary then corresponding secondary antibodies (Supplemental Table S2). Slides were mounted in ProlongDiamond with DAPI (Molecular probes). Imaging was performed using a Zeiss LSM 800 microscope using a Plan-Apochromat 63x/1.40 Oil objective.

## Supplementary information

**Additional file 1: Figure S1.** Colocalization between CSN and SMC5/6 components. (A) Co-localization of NSMCE4A and CSN5 in HeLa cells. (B) Relationship between the nucleocytoplasmic ratio of CSN5 and that of NSMCE4A. The plot indicates that the lower nucleocytoplasmic ratio of CSN5 corresponds to lower nucleocytoplasmic ratio of NSMCE4A. Pearson correlation coefficient: 0.718. (C-J) Colocalization of NSMCE1 with CSN1 (C), CSN2 (D), CSN3 (E), CSN4 (F), CSN5 (G), CSN6 (H), CSN7 (I), and CSN8 (J). (K-M) siRNA-mediated depletion of GPS1 does not affect the nucleocytoplasmic ratio or localization of NSMCE4A. (K and L) Representative images of siRNA control (siCTR) and siRNA-mediated depletion of GPS1 immunostained for NSMCE4A and GPS1. (M) The plot indicates that GPS1 siRNA treatment did not alter the nucleocytoplasmic distribution of NSMCE4A. (N and O) Treatment with CSN5i-3 does not affect the nucleocytoplasmic ratio of NSMCE4A. (N) representative images of cells untreated (NT) or treated with CSN5i-3 and immunostained for NSMCE4A and GPS1. (O) The plot indicates that CSN5i-3 (CSNi) treatment did not alter the nucleocytoplasmic distribution of NSMCE4A. Size bars = 10 μm

**Additional file 2: Figure S2**. Quantification of laser-induced DNA damage signal for γH2A.X and SMC6. Relative signal intensity increase at stripes compared to nuclear signal for γH2A.X (A) and SMC6 (B) for control (siCTR) and siRNA depletion of GPS1 (siGPS1). Relative signal intensity increase at stripes compared to nuclear signal for γH2A.X (C) and SMC6 (D) for control and CSN5i-3 treatments.

**Additional file 3: Figure S3.** Uncropped membranes and western blot images for GPS1 (A and B) and GAPDH (C and D) that are presented in Fig. 4.

**Additional file 4: Figure S4**. Uncropped membranes and western blot images for PARP1 (A and B) and Cul4a (C-E). Red boxes represent regions that are presented in Fig. 5.

**Additional file 5: Table S1**. Prey proteins that showed interaction with the bait NSMCE4A in yeast two-hybrid screening. The table shows the proteins which had at least one cDNA clone construct interacting with NSMCE4A bait on all the three-selection background (*AurA*, *−His*, *−Ade*). Subsequent verification after the screening was performed to minimize false positive hits. Secondary screening results indicated as “Yes” confirm a positive interaction result. Secondary screening results indicated as “No” failed to confirm initial interaction screening result.

**Additional file 6: Table S2**. Primary and secondary antibodies used in this study.

## Data Availability

Data and materials will be made available upon request via email to corresponding author (pjordan8@jhu.edu).

## Abbreviations

SMC: Structural maintenance of chromosomes
NSMCE: Non-structural maintenance of chromosomes element
GPS1: G protein pathway suppressor 1
COP9: Constitutive photomorphogenesis 9
CSN: COP9 signalosome
