# Supplementary figures and images for: Interaction between NSMCE4A and GPS1 links the SMC5/6 complex to the COP9 signalosome
Source: BMC Mol Cell Biol. 2020 May 8;21:36. doi: 10.1186/s12860-020-00278-x (PMC7206739; doi:10.1186/s12860-020-00278-x)

**Supplemental Figure S1**

**A**

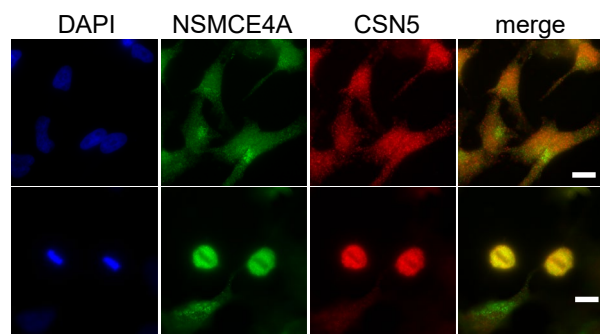

**B**

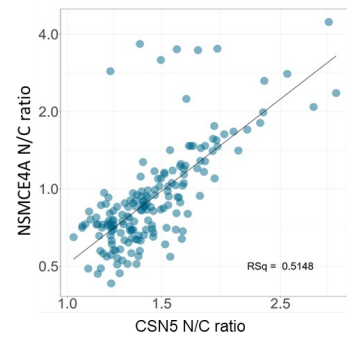

**C**

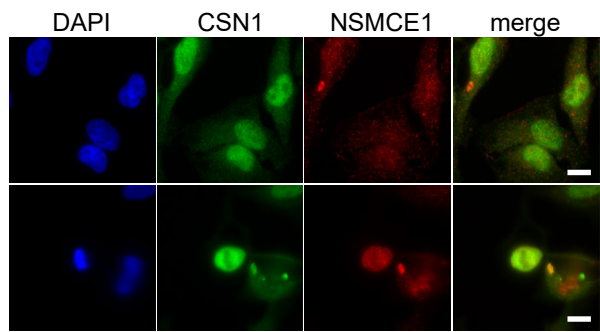

**D**

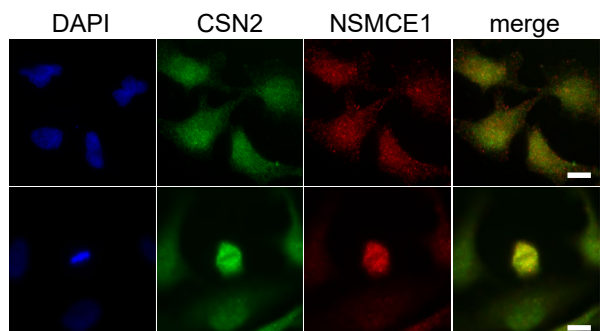

**E**

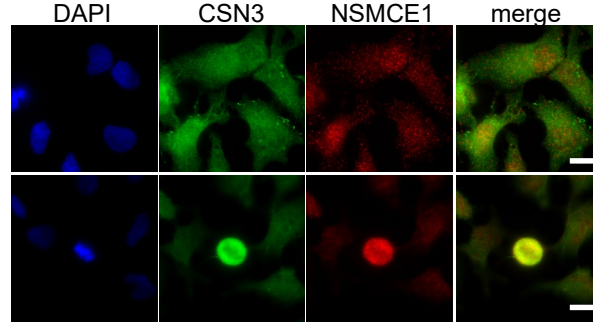

**F**

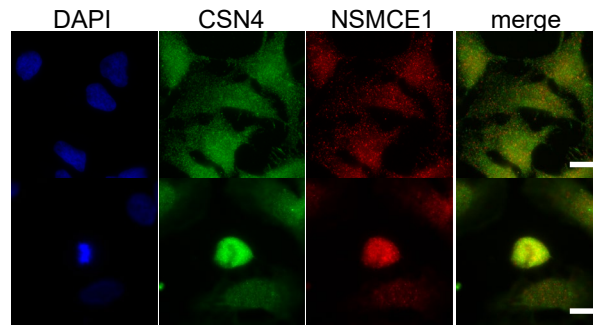

**G**

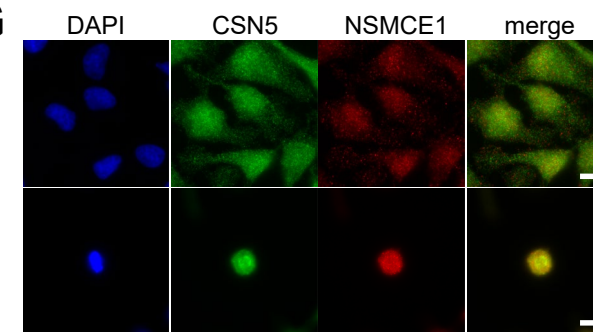

**H**

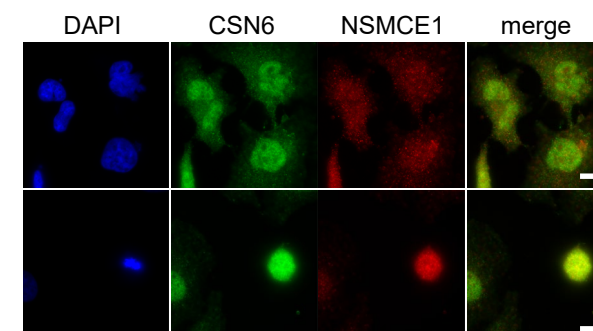

**I**

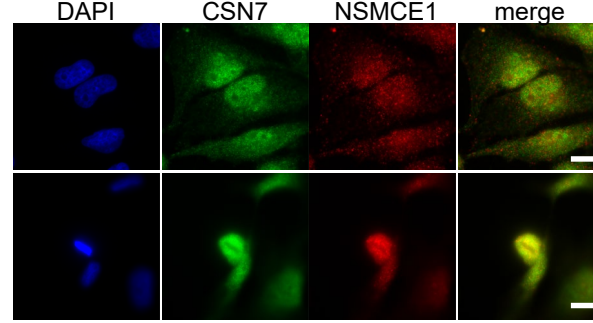

**J**

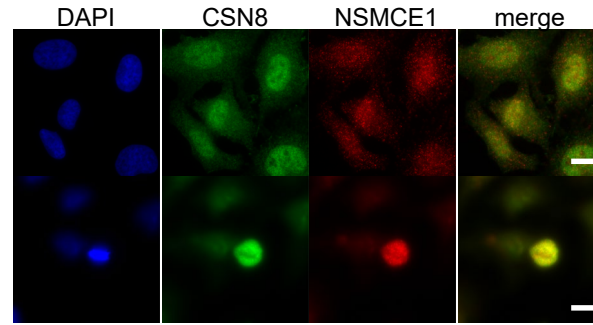

**K**

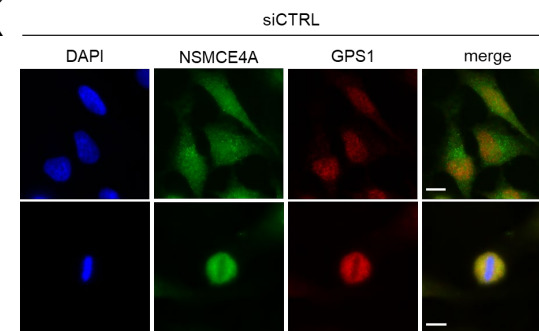

**L**

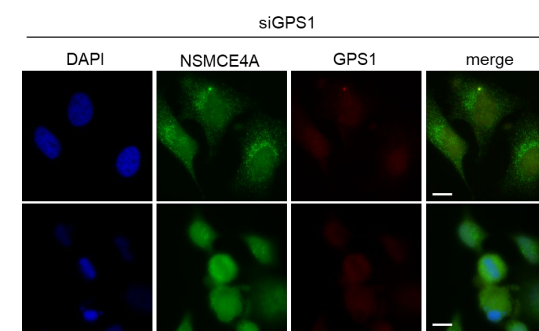

**M**

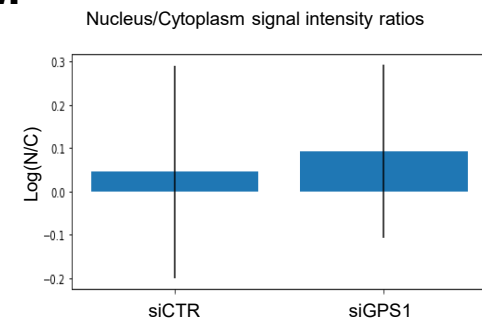

**N**

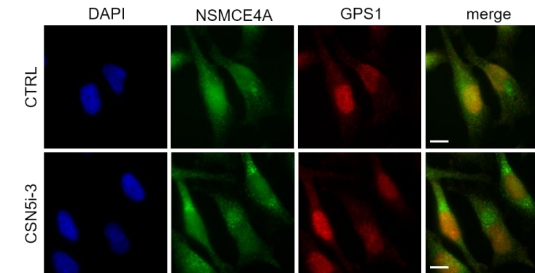

**O**

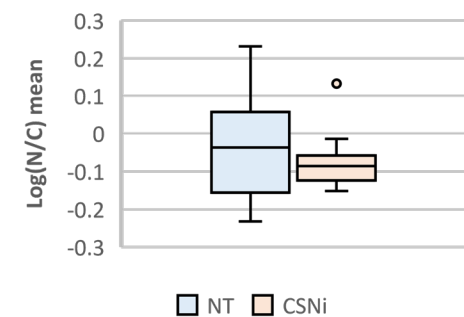

Supplement: Supplementary file 1 — Additional file 1: Figure S1. Colocalization between CSN and SMC5/6 components. (A) Co-localization of NSMCE4A and CSN5 in HeLa cells. (B) Relationship between the nucleocytoplasmic ratio of CSN5 and that of NSMCE4A. The plot indicates that the lower nucleocytoplasmic ratio of CSN5 corresponds to lower nucleocytoplasmic ratio of NSMCE4A. Pearson correlation coefficient: 0.718. (C-J) Colocalization of NSMCE1 with CSN1 (C), CSN2 (D), CSN3 (E), CSN4 (F), CSN5 (G), CSN6 (H), CSN7 (I), and CSN8 (J). (K-M) siRNA-mediated depletion of GPS1 does not affect the nucleocytoplasmic ratio or localization of NSMCE4A. (K and L) Representative images of siRNA control (siCTR) and siRNA-mediated depletion of GPS1 immunostained for NSMCE4A and GPS1. (M) The plot indicates that GPS1 siRNA treatment did not alter the nucleocytoplasmic distribution of NSMCE4A. (N and O) Treatment with CSN5i-3 does not affect the nucleocytoplasmic ratio of NSMCE4A. (N) representative images of cells untreated (NT) or treated with CSN5i-3 and immunostained for NSMCE4A and GPS1. (O) The plot indicates that CSN5i-3 (CSNi) treatment did not alter the nucleocytoplasmic distribution of NSMCE4A. Size bars = 10 μm [file 12860_2020_278_MOESM1_ESM.pdf]

## Slide 1
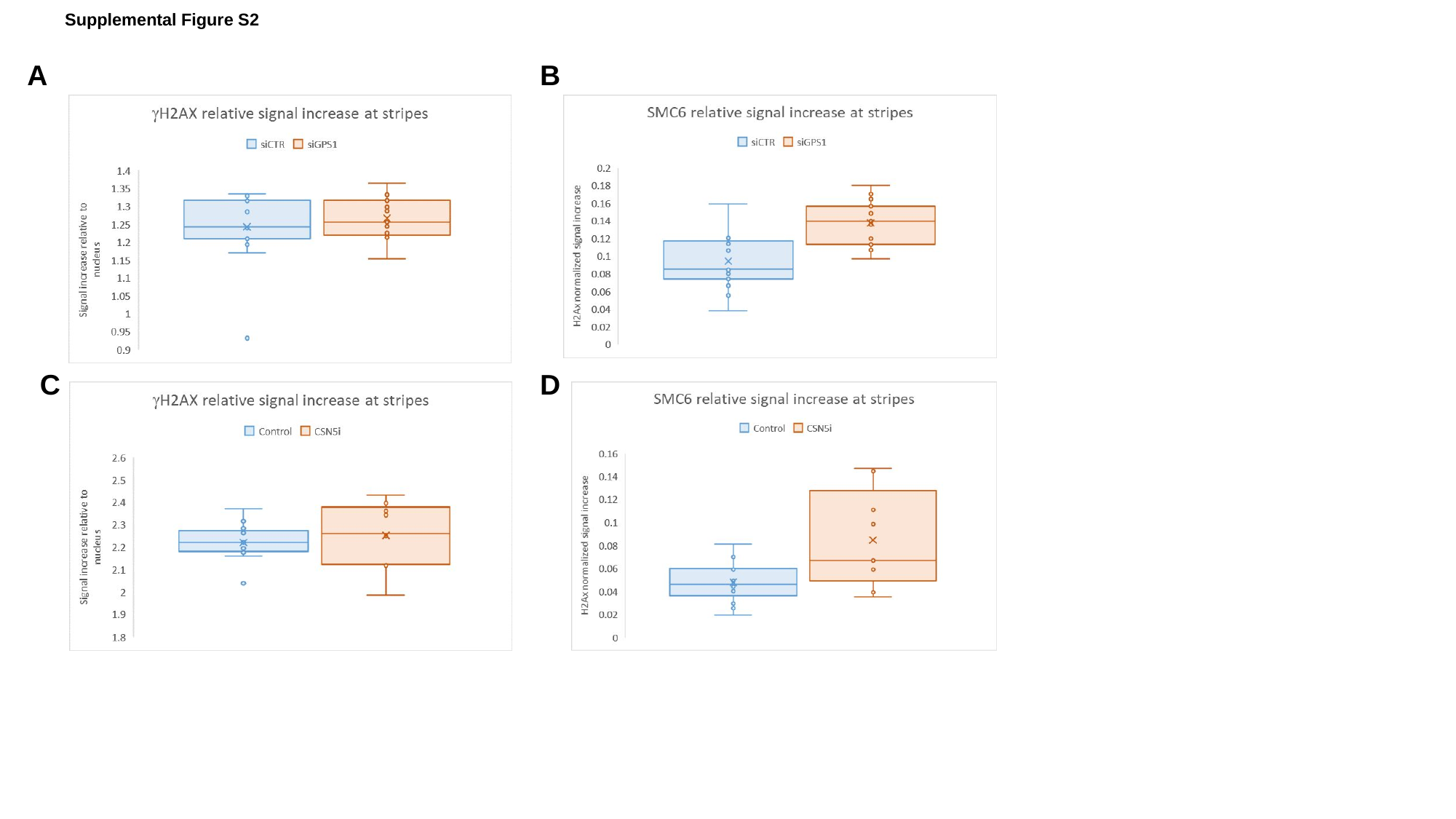

Supplemental Figure S2
A
B
C
D

Supplement: Supplementary file 2 — Additional file 2: Figure S2. Quantification of laser-induced DNA damage signal for γH2A.X and SMC6. Relative signal intensity increase at stripes compared to nuclear signal for γH2A.X (A) and SMC6 (B) for control (siCTR) and siRNA depletion of GPS1 (siGPS1). Relative signal intensity increase at stripes compared to nuclear signal for γH2A.X (C) and SMC6 (D) for control and CSN5i-3 treatments. [file 12860_2020_278_MOESM2_ESM.pptx]

Supplemental Figure S3

**A**

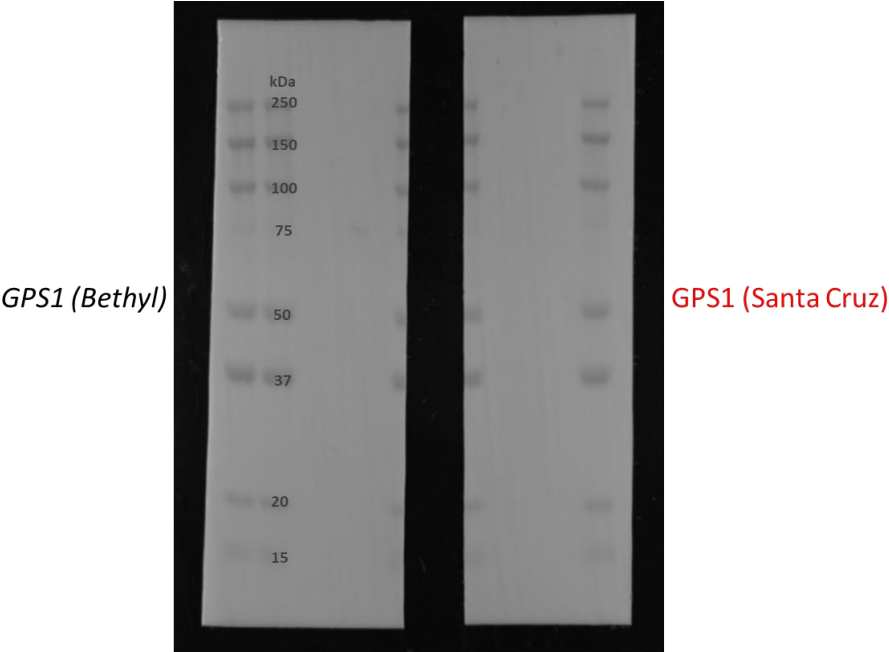

**B**

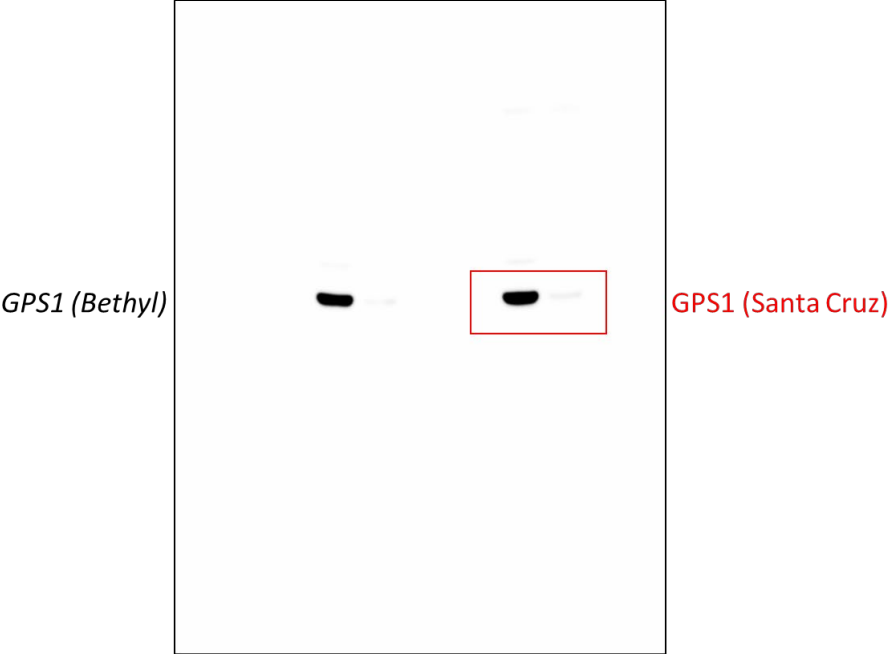

**C**

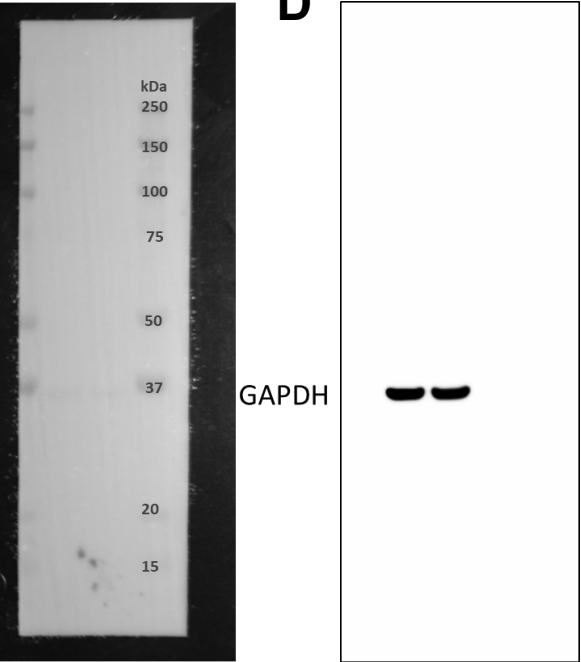

**D**

Supplement: Supplementary file 3 — Additional file 3: Figure S3. Uncropped membranes and western blot images for GPS1 (A and B) and GAPDH (C and D) that are presented in Fig. 4. [file 12860_2020_278_MOESM3_ESM.pdf]

Supplemental Figure S4

A

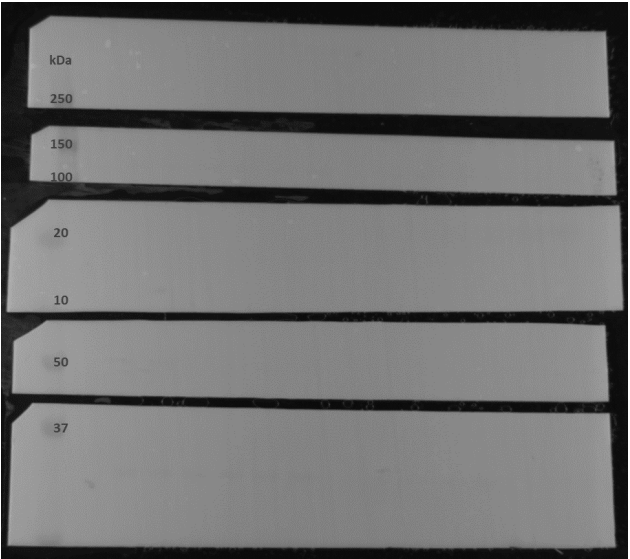

B

PARP1

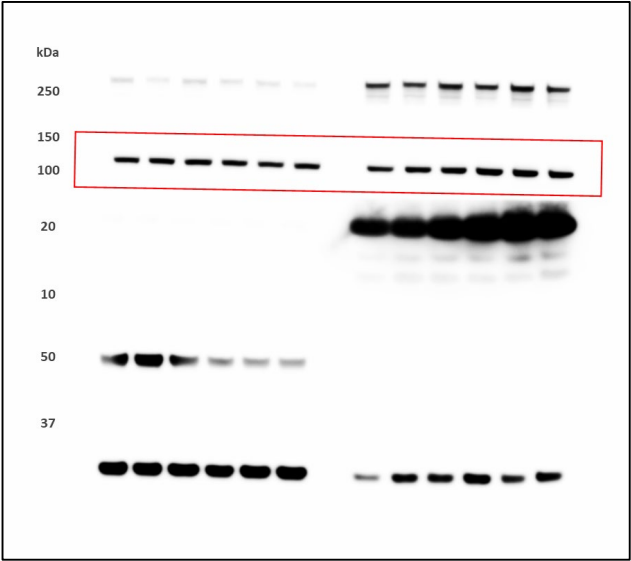

C

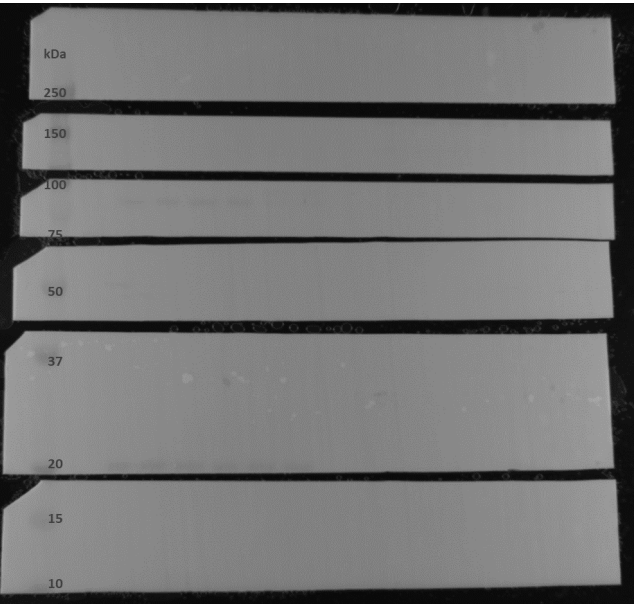

D

Cul4a

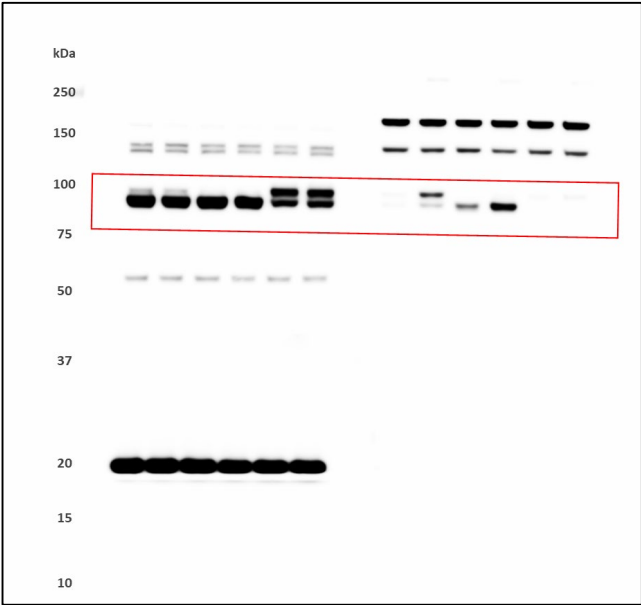

E

Cul4a

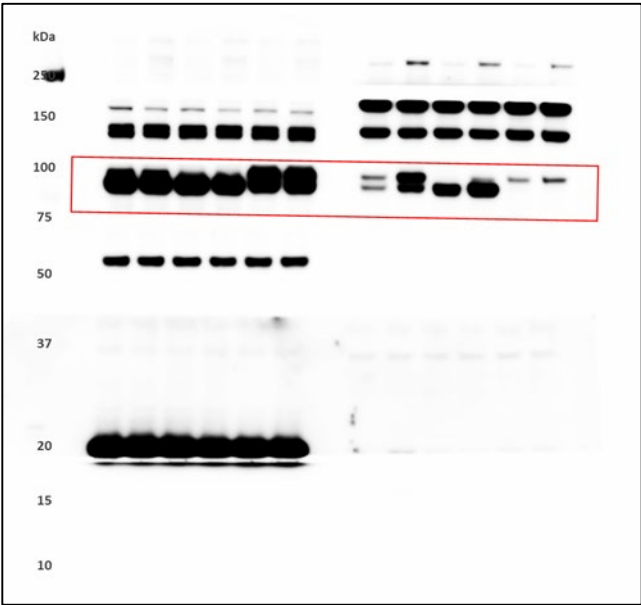

Supplement: Supplementary file 4 — Additional file 4: Figure S4. Uncropped membranes and western blot images for PARP1 (A and B) and Cul4a (C-E). Red boxes represent regions that are presented in Fig. 5. [file 12860_2020_278_MOESM4_ESM.pdf]
