# Supplementary material for: Interaction between NSMCE4A and GPS1 links the SMC5/6 complex to the COP9 signalosome
Source: BMC Mol Cell Biol. 2020 May 8;21:36. doi: 10.1186/s12860-020-00278-x (PMC7206739; doi:10.1186/s12860-020-00278-x)
Supplement: Supplementary file 5 — Additional file 5: Table S1. Prey proteins that showed interaction with the bait NSMCE4A in yeast two-hybrid screening. The table shows the proteins which had at least one cDNA clone construct interacting with NSMCE4A bait on all the three-selection background (AurA, −His, −Ade). Subsequent verification after the screening was performed to minimize false positive hits. Secondary screening results indicated as “Yes” confirm a positive interaction result. Secondary screening results indicated as “No” failed to confirm initial interaction screening result. [file 12860_2020_278_MOESM5_ESM.pdf]

Supplemental Table S1: Yeast two-hybrid bait interactions with NSMCE4A

Prey proteins that showed interaction with the bait NSMCE4A in yeast two-hybrid screening. The table shows the proteins which had at least one cDNA clone construct interacting with NSMCE4A bait on all the three-selection background (AurA, -His, -Ade). Subsequent verification after the screening was performed to minimize false positive hits. Secondary screening results indicated as "Yes" confirm a positive interaction result. Secondary screening results indicated as "No" failed to confirm initial interaction screening result.

Isolate ID	Protein ID	NCBI accession number	Positive secondary screen
NSM 4<br>NSM55<br>NSM61	RANBP9<br>RANBP9<br>RANBP9	https://www.ncbi.nlm.nih.gov/nuccore/NM_019930	Yes
NSM 5<br>NSM 111	NUP62<br>NUP62	https://www.ncbi.nlm.nih.gov/nuccore/NM_053074	No
NSM 7	SNAPIN	https://www.ncbi.nlm.nih.gov/nuccore/NM_133854.3	No
NSM 10	4930548H24RIK	https://www.ncbi.nlm.nih.gov/nuccore/NM_026296.3	Yes
NSM 12<br>NSM 56<br>NSM 98	Selenoprotein V<br>Selenoprotein V<br>Selenoprotein V	https://www.ncbi.nlm.nih.gov/nuccore/NM_175033	Yes
NSM 14	ASRGL1	https://www.ncbi.nlm.nih.gov/nuccore/NM_025610.3	Yes
NSM 15	COPS5	https://www.ncbi.nlm.nih.gov/nuccore/NM_013715.2	No
NSM 17<br>NSM 78	Epb41l4a<br>Epb41l4a	https://www.ncbi.nlm.nih.gov/nuccore/NM_013512.2	Yes
NSM 20<br>NSM 56	KIF9<br>KIF9	https://www.ncbi.nlm.nih.gov/nuccore/NM_001163569.1	Yes
NSM 21	GM5382	https://www.ncbi.nlm.nih.gov/nuccore/NM_001034100.1	Yes
NSM 23<br>NSM 63	SPATA4<br>SPATA4	https://www.ncbi.nlm.nih.gov/nuccore/NM_133711	No
NSM 25<br>NSM 50	SPATA3<br>SPATA3	https://www.ncbi.nlm.nih.gov/nuccore/NM_027300.3	Yes
NSM 53<br>NSM 99<br>NSM 84	GPS1<br>GPS1<br>GPS1	https://www.ncbi.nlm.nih.gov/nuccore/NM_145370	Yes
NSM59	CEP95	https://www.ncbi.nlm.nih.gov/nuccore/NM_177088.3	No
NSM 60	1700013624RIK	https://www.ncbi.nlm.nih.gov/nuccore/NM_027063.2	Yes
NSM 62<br>NSM 116<br>NSM 70	ItgB3BP<br>ItgB3BP<br>ItgB3BP	https://www.ncbi.nlm.nih.gov/nuccore/NM_026348	Yes
NSM 110	NAA50	https://www.ncbi.nlm.nih.gov/nuccore/NM_028108.3	No
NSM 115<br>NSM 112	MED10<br>MED10	https://www.ncbi.nlm.nih.gov/nuccore/NM_138596.2	Yes
NSM 100	TNNI3	https://www.ncbi.nlm.nih.gov/nuccore/NM_009406.4	Yes
NSM 64	CHAMP1	https://www.ncbi.nlm.nih.gov/nuccore/NM_001363455.1	Yes
NSM 117	SYCE1	https://www.ncbi.nlm.nih.gov/nuccore/NM_001143765.1	Yes
NSM 96	CCDC24	https://www.ncbi.nlm.nih.gov/nuccore/NM_001034876.1	No
NSM 77	ABT1	https://www.ncbi.nlm.nih.gov/nuccore/NM_013924.3	No
NSM 65	PpfibP1	https://www.ncbi.nlm.nih.gov/nuccore/NM_001355729.1	Yes
NSM 103	TXNDC2	https://www.ncbi.nlm.nih.gov/nuccore/NM_001146002.1	No
NSM 93	DNAJB6	https://www.ncbi.nlm.nih.gov/nuccore/NM_001359198.1	No
