# Supplementary material for: Interaction between NSMCE4A and GPS1 links the SMC5/6 complex to the COP9 signalosome
Source: BMC Mol Cell Biol. 2020 May 8;21:36. doi: 10.1186/s12860-020-00278-x (PMC7206739; doi:10.1186/s12860-020-00278-x)
Supplement: Supplementary file 6 — Additional file 6: Table S2. Primary and secondary antibodies used in this study. [file 12860_2020_278_MOESM6_ESM.pdf]

Supplemental Table S2: Antibodies used in this study

Primary Antibodies

Antibody target	Host	Source	Cat. Number	IF Dilution	WB Dilution
NSMCE4A	Rabbit	Genetex	GTX121270	1:500	1:1000
NSMCE1	Mouse	Abcam	ab168578		1:1000
GPS1	Mouse	Santa Cruz Biotechnology	sc-365617	1:100	1:1000
CSN5	Mouse	Santa Cruz Biotechnology	sc-13157	1:100	
CSN3	Rabbit	Bethyl Laboratories	A300-012A		1:1000
GPS1	Rabbit	Enzo Life Sciences	BML-PW8285	1:250	
CSN2	Rabbit	Enzo Life Sciences	BML-PW8230	1:250	
CSN3	Rabbit	Enzo Life Sciences	BML-PW8235	1:250	
CSN4	Rabbit	Enzo Life Sciences	BML-PW8360	1:250	
CSN5	Rabbit	Enzo Life Sciences	BML-PW8365	1:250	
CSN6	Rabbit	Enzo Life Sciences	BML-PW8295	1:250	
CSN7	Rabbit	Enzo Life Sciences	BML-PW8300	1:250	
CSN8	Rabbit	Enzo Life Sciences	BML-PW8290	1:250	
SMC6	Rabbit	Abcam	ab18039	1:500	
γH2A.X	Mouse	Millipore	05–636	1:2000	
CUL4A	Rabbit	Bethyl Laboratories	A300-739A		1:1000
PARP1	Rabbit	Cell Signaling Technology	9542S		1:2000
GAPDH	Mouse	Sigma	G8795		1:5000
HA	Rabbit	Origene	TA150034	1:200	1:1000
FLAG	Mouse	Sigma	F3165	1:2000	1:10,000

Secondary Antibodies

Antibody	Host	Source	Cat. Number	IF Dilution	WB Dilution
Mouse IgG (H+L), Alexa Fluor 488	Goat	Invitrogen	A-11001	1:500	
Mouse IgG (H+L), Alexa Fluor 555	Goat	Invitrogen	A28180	1:500	
Mouse IgG (H+L), Alexa Fluor 568	Goat	Invitrogen	A-11031	1:500	
Rabbit IgG (H+L), Alexa Fluor 488	Goat	Invitrogen	A-11008	1:500	
Rabbit IgG (H+L), Alexa Fluor 555	Goat	Invitrogen	A27039	1:500	
Rabbit IgG (H+L), Alexa Fluor 568	Goat	Invitrogen	A-11011	1:500	
Mouse IgG (H+L), HRP	Rabbit	Invitrogen	31450		1:20,000
Rabbit IgG (H+L), HRP	Goat	Invitrogen	31466		1:20,000
